# Comments on the April Issue of the Texas Medical Journal

**Published:** 1916-07

**Authors:** 


### Comments on the April Issue of the Texas Medical Journal.

THE TEXAS MEDICAL JOURNAL.—This publication for April was a pleasant surprise to us, for it was a real "Woman's Issue." Its talented and successful editor, Mrs. F. E. Daniel, Austin, Texas, has made this issue one of the brightest and best of any of our exchanges so far received, and the unusual part of it is that every one of its original articles are contributed by women physicians. We congratulate you, Mrs. Daniel, with all of our heart, for this demonstration of the abilities of your brilliant editorial staff. We are proud of you as a representative of Southern medical journalism.—*Southern Clinic.*

---

### DUX FEMINA FACTI.

The publisher and managing editor of the TEXAS MEDICAL JOURNAL—the well known Red Back—is a clever woman, Mrs. F. E. Daniel. A sisterly instinct impelled her to fill the April issue of her admirable publication with original articles by women physicians, and the showing is creditable and interesting.

Dr. Ray Karchmer Daily writes on "Vincent's Angina," Dr. Mary Cleveland Harper on "Difficulties in Breast Feeding," Ethel Lyon Heard on "Clean Milk," Dr. Violet H. Keiller on "Renal Tuberculosis," Dr. Minnie L. Maffett on "Abnormal Delinquency," and Dr. Martha A. Wood on the "Blood Smear in Diagnosis."—*New York Medical Journal.*

